# On finding one’s way: a comment on Bock et al. (2024)

**DOI:** 10.1007/s00426-024-02011-1

**Published:** 2024-07-25

**Authors:** Carl T. Woods, Duarte Araújo, Keith Davids

**Affiliations:** 1 Institute for Health and Sport, Victoria University, Melbourne, Australia; 2 Faculdade de Motricidade Humana, CIPER, Universidade de Lisboa, Cruz Quebrada, Lisbon, Portugal; 3 Sport and Human Performance Research Group, Sheffield Hallam University, Sheffield, UK

## Abstract

In a recent issue of *Psychological Research*, Bock, O., Huang, J-Y., Onur, O. A., & Memmert, D. (2024). The structure of cognitive strategies for wayfinding decisions. *Psychological Research Psychologische Forschung*, *88*, 476–486. 10.1007/s00426-023-01863-3.) investigated cognitive strategies purported to guide wayfinding decisions at intersections. Following experimentation in a virtualised maze, it was concluded that intersectional wayfinding decisions were based on a ‘generalized cognitive process’, in addition to ‘strategy-specific’ processes. The aim of our comment is not to challenge these findings or their methodological rigour. Rather, we note how the study of human wayfinding has been undertaken from entirely *different* metatheoretical perspectives in psychological science. Leaning on the seminal work of James Gibson and Harry Heft, we consider wayfinding as a continuous, integrated perception-action process, distributed across the entire organism-environment system. Such a systems-oriented, ecological approach to wayfinding remediates the organismic asymmetry pervasive to extant theories of human behaviours, foregrounding the possibility for empirical investigation that takes seriously the socio-cultural contexts in which inhabitants dwell.

## Introduction

How do animals – humans in the present case – find their way around the environment? This profound question extends across many scientific disciplines, and its response, as we seek to highlight here, is far from transparent. In a recent issue of *Psychological Research*, Otmar Bock and colleagues (2024) sought to address such a question, framing their investigative focus on cognitive strategies purported to guide wayfinding decisions at intersections. We read this work with interest and after careful consideration, were encouraged to write a comment. Though, the seed of this encouragement was not to draw criticism toward the target article. Rather, we considered the commentary as an opportunity generate a humble point of contrast, offering readers of *Psychological Research* an entirely *different* perspective as to how humans – as active perceivers – find their way through structured environments inhabited with and alongside others. We aim to do this across two sections.

Section one opens by exploring the *interactionist* grounding pervasive to much psychological research on human wayfinding. As a metatheory, interactionism starts from the implicit assumption and commitment to conceptualising the individual as a bounded, independent and semi-autonomous entity that exists in a surround, separate to other independently bounded entities (Heft, 2001, 2013a). This perspective fosters a mechanistic and linear view of causality, most noted through input-output models of behaviour (Heft, 2001). A manifestation of these models is a unit of analysis scaled, not just to the bounded individual, but at the level of cognitive processes purported to operate therein (Dunwoody, 2006; Davids & Araújo, 2010). Here, we briefly discuss how interactionism has shaped the study of human wayfinding in psychological science, situating the target article amongst this tradition. Note, this is not in criticism of the article’s rigour, but to foreground a point of metatheoretical distinction and departure in discussing wayfinding.

In section two, we lean on the seminal work of James Gibson and Harry Heft to discuss wayfinding as an *ecological* phenomenon, facilitated by the direct perception of environmental information over time. Not only does this perception-action approach foreground a temporal dimension omitted by interactionist accounts of wayfinding, but – in keeping with the metatheory of *transactionism* – it distributes control across the organism-environment system. These considerations are important for two reasons. First, it remediates the *organismic asymmetry* germane to extant theories of wayfinding rooted in an interactionist framework. Second, it allows us to consider wayfinding as a temporally structured skill, which points toward the need for empirical investigation that takes seriously the socio-cultural contexts in which inhabitants dwell (Araújo et al., 2019; Heft, 2013a, b; Hutchins, 1995; Ingold, 2000). Ultimately, we hope our comment not only generates a point of relevant contrast to ideas proposed by Bock et al. (2024), but offers readers of *Psychological Research* with an entirely different metatheoretical grounding from which to explore the phenomenon of wayfinding.

## Section 1: from a bird’s-eye view…

In science, a theoretical approach can broadly be viewed as belonging to a family of theories that share common *metatheoretical* assumptions and commitments. By metatheoretical, we mean basic, and oftentimes implicit, tenets that relate to features of a particular theory. Indeed, the eminent philosopher-scientist Juarerro (2023) referred to the assumptions that scientists make – when adopting a specific theoretical framework to guide their conceptualisation, planning and research designs – as ‘apparent’. These assumptions may only be ‘apparent’ because some scientists may not be fully aware of the metatheoretical commitments that accompany a particular theory. Operating at high levels of abstraction, these commitments typically frame presuppositions related to the primary subject matter addressed (see Heft, 2012). Of particular relevance to 20th -century environmental psychology are the four metatheories outlined by Altman and Rogoff (1987): the *trait* metatheory, the *interactionist* metatheory, the *organismic* metatheory, and the *transactional* metatheory (also see Heft, 2012). It is the interactionist metatheory that is of relevance to this section, given its pervasiveness to the study of human wayfinding in psychological science (for an overview, see Heft, 2013a).

Interactionism starts from the implicit assumption that the individual is a bounded entity existing in a surround, independent to other (independently) bounded entities (Heft, 2012). In psychological science, this assumption typically manifests in three interrelated features. First, the *unit of analysis* is scaled to the individual, viewed as a semi-autonomous entity that exists separate to a surround, yet is still open to ‘outside’ influences (Heft, 2001, 2012). Second, the *source of change* in an individual’s organisational state resides beyond the psychological domain, either in the environment or in biological predispositions (Heft, 2001, 2012). As a brief note, a common manifestation of this second feature are information-processing models that imbue linear sequences of causation – that is, an input stimulus from the environment is processed in the mind, which leads to an outputted response by the body. Third, *causal influences* are seen to operate locally and linearly, in a chain-like fashion (Heft, 2013a). Such is the pervasiveness of this ‘efficient causality’ that most 20th -century psychology conflates the word ‘cause’ with a consequent ‘effect’ (Heft, 2012, p. 15).

### Interactionism and the study of human wayfinding

In an exceptional essay titled *Environment*,* Cognition and Culture: Reconsidering the Cognitive Map*, Heft (2013a) demonstrates how the ripples of interactionist thinking have (implicitly and continuously) shaped studies of human wayfinding in psychological science. Such work, according to Heft, tends to orient a combination of three extant perspectives: (i) an information-processing approach (Golledge, 1987), (ii) a Piagetian developmental tradition (Hart & Moore, 1973), and iii), a nativist approach, with deep ties to Cartesian and Kantian thought (Spelke et al., 2010). While an excursus of each is beyond the scope of this comment, it is important to note that each perspective shares at least one characteristic of interactionism. Namely, each perspective scales their unit of analysis to the individual. While directing some attention toward the environment, each conceptualises the nature of knowledge as specific to the individual, thereby epistemically separating individual processes from changes in environing conditions.

According to Dunwoody (2006), and later advanced by Davids and Araújo (2010), this unit of analysis is troublesome, as it perpetuates an *organismic asymmetry*. In psychological science, this distortion is detected through explanations of human behaviour that inherently bias internal or representational accounts at an expense of the organism-environment *relation*. Woven into the study of human wayfinding, this organismic asymmetry often leads to the view that survey knowledge and configurational understanding of the environment are critical forms of spatial knowing. It is our opinion that the target article perpetuates such an *organismic asymmetry*, noted most apparently in the opening paragraph when the authors describe how humans find their way:

We need to *process spatial information* from multiple sensory modalities, *maintain internal representations of the environment*, plan routes, make decisions at intersections, control our gait, monitor our position and heading in space, and *orchestrate these processes by overarching executive control*. (Bock et al., 2024, p. 476, emphasis added)

While the authors do not make explicit their metatheoretical grounding, such an opening statement (to us) can be interpreted as demonstrating the specific positioning of the article as (implicitly) wedded to interactionist thinking. To elaborate: the referral to ‘*process spatial information*’ clearly advocates an information-processing approach, which can be historically traced to Tolman’s (1946, 1948) neo-behaviourist work on the cognitive map. This stance posits that individuals acquire ‘expectations’ from prior experiences, which are believed to facilitate behavioural choices and bias deliberations when faced with (unexpected) changes in an environmental layout. As the header of this section points toward, a common conceptualisation of these acquired expectations is survey knowledge about the environment, where a layout perspective is not located on the ground, but rather atop, from a drone-like, ‘birds-eye view’ (Heft, 2013a, p. 18). Second, the referral to ‘*maintain internal representations of the environment*’ acknowledges a nativist approach, which has deep ties to Cartesian and Kantian thought. As Heft (2013a, p. 18) shows, representational claims of spatial knowing speak, not to spatial relations derived from first-hand experience, but to spatial understandings that arguably *presuppose* them. This perspective leads to the third point: the referral to wayfinding processes being orchestrated by ‘*overarching executive control*’. Such a statement not only speaks to an ensuing body-environment and mind-body dualism, but it foregrounds the Piagetian tradition in its emphasis on presupposed mental processes believed to be in place from the outset (Heft, 2013a, p. 18).

Taking a brief step back, relevant questions to pose at this point are: Do psychological scientists interested in human movement and sports performance, need to address such metatheoretical concerns at all? In other words, why is it important to highlight the specificity of the implicated beliefs that underpin the conceptualisation chosen to frame experiments, like that of Bock et al. (2024)? In response, it is worth noting how different metatheoretical positions oft-incorporate different conceptualisations of causality, leading to different forms of explanation (Stepp et al., 2011). This means that our metatheoretical orientation not only (implicitly) guides subsequent empirical investigations related to a phenom of concern, *it frames the phenom of concern*. Considered in the study of human wayfinding, a prevailingly interactionist orientation commits researchers to representational accounts of behaviour, as it starts from the assumption that individuals exist *separate* to a surround. Thus, from the interactionist grounding Bock and colleagues (implicitly) stake out, it would appear completely sensical to investigate various cognitive strategies purported to guide wayfinding decisions at intersections, as the unit of analysis was (implicitly) scaled to the individual from the outset – or more specifically, the ‘overarching executive’ functions operating therein. Perhaps this is one of the reasons why the decision-making task used by Bock and colleagues was virtual, housed to a computer screen: i.e., because the authors implicitly follow principles of a metatheory comfortable with disembodying bounded, passive individuals in their deliberations, detaching them from the environment of which they constitute was of little concern. In this interactionist approach, there is little need to consider how the participants’ exploratory movements – nested in an environment they could actually *see*, *hear*, *smell*, *touch* and *taste* – implicated how they learnt to find their way. So say the authors:

The participants were asked to follow a *prescribed route* through a virtual maze that was presented on a computer screen. They were *transported passively* to an intersection of corridors, where they stopped. Then they had 3000 ms to indicate whether the route continued straight on, to the left or the right, by *deflecting the handle of a joystick* in the pertinent direction. They could respond anytime during the 3000-ms interval, ad [sic] were not rushed to do this quickly. (Bock et al., 2024, p. 478, emphasis added)

To reiterate the intentions of this commentary, our concern is not to criticise the authors for their metatheoretical orientation (assumed or otherwise), nor their subsequent empirical investigation. As far as we could see, the assumptions and commitments of an interactionist metatheory were impeccably followed in their study. Rather, we simply note that wayfinding can be conceptualised from an entirely *different* metatheoretical perspective: a non-representational account scaled, not to the (executive level of a) bounded individual, but to the person-environment relation. Stated differently, what if – contrary to the assertions of Bock et al. (2024) – wayfinding is not orchestrated by ‘overarching executive’ functions, but is a process distributed across the complex, organism-environment system, facilitated by the direct perception of environmental information over time? It is thus here, where we flag our point of metatheoretical departure.

## Section 2: …to a path of observation

Recall earlier, where we introduced the four metatheories outlined by Altman and Rogoff (1987). Our commentary, thus far, leads us to the *transactional* metatheory, which we would like to pay attention to now. Differing to the assumptions of interactionist thinking, transactionism scales its foundational unit of analysis to the organism-environment system; the components of which are seen to operate in a *relationally interdependent* way (Heft, 2012, p. 16). This relationality means it subverts the underlying *organismic asymmetry* germane to extant theories of interactionism by adopting the view that psychological processes and experiences are mutually anchored in *both* environmental features and an organism’s action capabilities (Heft, 2021, p. 241). The organism and environment, in other words, constitute one another – they are inseparable from the outset. Taking this transactional grounding as our basis, we can now start to explore extant theories of wayfinding that focus on this relational scale of analysis.

### Toward an ecological approach to visual perception

To initiate this exploration, we turn to the work of perceptionist, James Gibson. In the 1960s and 1970s, Gibson developed an ecological approach to psychology that departed from the (still dominant) mentalist tradition. In its radical departure, Gibson focused attention on the nature of perceiving, which can be broadly denoted through three major tenets: first, perception is a *directly active process*; second, perception is for the *guidance of action*; third, perception is of *affordances* (see also Gibson, 1988; Heft, 2001; Reed, 1996; Chemero, 2009). In accord with commitments of transactionism, these tenets start from the premise that the animal and environment are inseparable; each implies the other. This, after all, is what makes Gibson’s approach radically *ecological*. While a detailed excursus of each tenet is beyond the scope of our comment, it is within our bounds to briefly discuss the first two tenets, given their implications on forthcoming discussion.

To open, it is notable that Gibson’s ecological approach is fundamentally rooted in direct or ecological realism (Gibson, 1967; see also Shaw et al., 1982; Turvey & Carello, 1981; Turvey, 2018). To say that perception is ‘direct’, then, is to say that it does not involve internal representation – the world is ‘here’, directly accessible to an active perceiver (Gibson, 1979/2014). As anthropologist Ingold (2000) puts it: “the skilled practitioner consults the world, rather than representations (rules, propositions, beliefs) inside his or her head, for guidance on what to do next” (p. 164). This means that ‘active’ does not encapsulate a mental process of predicting, deliberating, inferring or representing, but quite literally refers to movement: of eyes, head, torso and body in finding and using information that envelops us all. In speaking to this directly active account of perception, philosopher Noë (2015) writes:

The scientist’s conception is impoverished, and it gets in the way of our appreciating that it is not brains that perceive, *but active animals or people*. Seeing […] is more like climbing a tree, or reading a book, than it is digesting what you’ve eaten. (p. xii, emphasis added)

In less metaphoric prose, as an animal moves about, they detect invariant information in an ambient array that directly specifies an environment’s layout *in relation* to their individual point of observation (Gibson, 1979/2014). Movement produces a changing array of stimulation, which makes invariant information specific to environmental features easier to detect (Gibson, 1988). This leads us to the second tenet discussed here: perception not only guides action, but action reciprocally guides perception (Gibson, 1979/2014; also see Chemero, 2009; Heft, 2012; Reed, 1996). Contrary to extant theories germane to interactionism, perception and action are not separable functions within an ecological approach; they constitute a jointly operative *perceptual system* (Gibson, 1966). This coupling holds that action is not controlled or orchestrated by overarching executive functions, but:

[…] by information, that is, by seeing oneself in the world. Control lies in the *animal-environment system*. Control is by the animal *in* its world, the animal itself having subsystems for perceiving the environment and concurrently for getting about in it. (Gibson, 1979/2014, p. 215, emphasis added and in original)

An important aspect of this perception-action coupling is that it speaks to temporal dimension that is oft-omitted from interactionist accounts of behaviour, owing (in part) to an underlying assumption that vision is an image-capturing process (Heft, 2013a). Given its grounding in direct realism, Gibson’s ecological approach does not follow such an assumption. Rather, animals see the environment *during* locomotion, not just in the pauses along the way. That is, they see – as the header of this section points toward – along a *path of observation* (Gibson, 1979/2014, p. 187).

Before progressing, it is worth briefly contrasting this temporal component with the decision-making task used by Bock et al. (2024). Recall in the description of this task, participations were given 3000-ms to decide which way to move, deflecting a joystick in the pertinent direction. Not only does this methodology speak to vision as an image-capturing process, but it underscores a two-stage deliberation cycle: that is, participants first visually snap-shot the environment, and then following a processing period, a response is outputted by a passive body. The deliberation in this decision-making task, in short, could be thought of as the connecting up of a finite set of adjacent points from afar (cf. Araújo et al., 2006; Araújo et al., 2014; Correia et al., 2012). In accord with the ecological approach to visual perception advocated for here, a path of observation need not be treated as such. Rather, it can be thought of as a *unitary movement* that unfolds over short (i.e., minutes, hours, days, weeks) or long (i.e., months, years, decades) periods. Taking this perception-action approach as our basis, we can now explore an ecological approach to human wayfinding.

### Wayfinding as an ecological phenom

In his magnum opus, *The Ecological Approach to Visual Perception*, Gibson advanced an explanation of human wayfinding by first rejecting the dominant interactionist grounding pervasive to current explanations:

Way-finding is surely not a sequence of turning responses conditioned to stimuli. But neither is it the consulting of an internal map of the maze, for who is the internal perceiver to look at the map? The *theory of reversible occlusion* can provide a better explanation. (Gibson, 1979/2014, p. 189, emphasis added)

In short, Gibson’s theory of reversible occlusion proposes that humans find their way in terms of the specific order in which the surfaces of the environment come into and out of sight as they proceed along a path of observation. To elaborate, let us suppose we are walking along a street in a city, perhaps finding our way toward a university to attend a research symposium. Notwithstanding the sounds we hear, the wind we feel, or perhaps the pollutants we smell, the surfaces we see – the facades of the buildings and the rising pavement beneath our feet – comprise what Gibson (1979/2014, p. 189) calls a “*vista*”. A vista can be thought of as “a semienclosure, a set of unhidden surfaces […] what is seen from here, with the proviso that ‘here’ is not a point but an extended region”. Let us now suppose that as we move down the street, we reach a corner, opening up a new vista, while occluding that of the previous. In accord with the theory of reversible occlusion, we have reached what Gibson (1979/2014, p. 189) refers to as a *transition*. Thus, to travel from place to place involves the opening up and closing off of vistas through a continuous series of reversible transitions.

There is an important point to note here, which is that in a terrestrial environment of semienclosed places, each vista is unique. It is its own ‘landmark’, inasmuch as habitats never duplicate (Gibson, 1979/2014). How humans learn to orient themselves, then, is through the structured ordering of vistas; a process learnt through *exploratory locomotion*. As Gibson states:

When the vistas have been put in order by exploratory locomotion, the invariant structure of the house, the town, or the whole habitat will be apprehended. The hidden and unhidden become one environment. One can then perceive the ground below the clutter out to the horizon, and at the same time perceive the clutter. One is oriented to the environment. It is not so much having a bird’s-eye view of the terrain as it is being everywhere at once. (ibid. p. 189)

Bringing this explanation back to the target article, we can now appreciate that the design of the decision-making task – which passively transported participants to intersections – would not suffice in accord with the theory of reversible occlusion. For among other things, it does not make room for the exploratory locomotion needed to detect the structure of the environment, guiding the ordering of vistas.

To this end, it is worth mentioning some key empirical developments to Gibson’s theory of reversible occlusion, noted most apparently through the seminal work of Harry Heft. Across a series of experiments, Heft (1979, 1983, 1996) showed that individuals traveling along a route through the environment are indeed sensitive to the ordering of vistas. Moreover, the transitions of these vistas are meaningful places for individuals, the value of which for wayfinding increases with repeated exposure (see Heft, 2013b). The importance of these insights cannot be overstated, for they point toward the *relational* character of information used for wayfinding. To elaborate, let us suppose we are traveling along a path toward a park bench, generating a pattern of optical flow as we go. The flow of this information is referred to as *perspective structure* (Gibson, 1979/2014), and changes as we travel along the path. The bench, which is positioned at the centre of an optic array, does not appear to move as we approach it (so long as our path remains straight), though our retinal image of it does increase in size as we get closer. That is, it *expands* (see Reed, 1996, p. 95). Comparatively, at the edge of the path, the grass and trees sweep by, until they pass out of sight behind us, converging around a point of *contraction* (see Reed, 1996, p. 95). These two poles of perspective structure, the focus of expansion and of contraction, are natural laws of locomotion, which – as this example shows – specify the observer’s movements relative to the layout of the environment (see also Warren, 2006). In other words, *the information used for wayfinding is actively generated reciprocally dependent of both observer and environment*. For this reason, Heft (1996) suggests that to find one’s way is to travel along a path so as to actively generate the flow of perspective structure distinct to the path which leads to one’s destination. Wayfinding, in this regard, could be thought of as a *temporally structured skill*, akin to humming a melody:

In music, a melodic phrase is not just a sequence of discrete tones; what counts is the rising or falling of pitch that gives shape to the phrase as a whole. Likewise in wayfinding, the path is specified not as a sequence of point-indexical images, but as the coming-into-sight and passing-out-of-sight of variously contoured and textured surfaces. (Ingold, 2000, p. 238/9)

To round this out, we can surmise an ecological approach to wayfinding in two distinct ways relative to that advanced by Bock et al. (2024). First, it is a *perception-action process* controlled by the detection of information over time, which directly specifies a route through a vista. Given its relational quality, this information is actively generated as an observer moves along, which is referred to as perspective structure. Second, because this information is direct, unequivocally specifying the route, there is no need to represent a surround. The environment is ‘(t)here’, to be directly perceived by an attentive observer. There is a corollary to this, briefly touched on next, which relates to the *skill* of wayfinding.

### Wayfinding as a temporally structured skill

Positioned ecologically, as a perception-action process that unfolds over time, wayfinding can be considered as a temporally structured, goal-directed skill. To elaborate, let us turn once again to Gibson (1966; 1979/2014), who drew a distinction between two general types of knowing: *knowing about* and *knowing of*. *Knowing about* refers to some state of affairs, such as knowing that the Melbourne Cricket Ground is located in Victoria, Australia, or that tomatoes are needed when making a lasagne. Comparatively, *knowing of* refers to skilful action that results in some desired outcome, such as cooking the tomatoes when making a lasagne, or actually finding your way to the Melbourne Cricket Ground without reverting to the assistance of navigational aids. The former type of knowing is information transmitted at second-hand, often produced by another human individual, while in the latter, information is gained primarily, by directly perceiving features of an environment for oneself. So says Gibson (1979/2014):

*Knowledge of* the environment…develops as perception develops, extends as the observers travel, gets finer as they learn to scrutinize, gets longer as they apprehend more events, gets fuller as they see more objects, and gets richer as they notice more affordances. Knowledge of this sort does not “come from” anywhere; it is got by looking, along with listening, feeling, smelling, and tasting. (p. 242, emphasis added)

The latter part of this excerpt points toward a *skilfulness* that grows out of action rather than explication. Unlike *knowing about*, which involves detaching oneself from action to assume a reflective stance (see Heft, 2013b), *knowing of* involves acting *with* features or properties of an environment in the achievement of a goal-directed outcome. Skilfully making a lasagne, for example, is a process that involves far more than merely reading a recipe from a cookbook. It requires one to *work with* the ingredients in a particular way (i.e., cooking, not burning them), and the subsequent utensils, which are all nested in a kitchen of varying ongoings, so as to produce a particular taste and texture that connotes a lasagne. Skilled action, in sum, is a body-environment process (Ingold, 2000).

Let us now consider this in the context of wayfinding (see also Woods et al., 2020). As established in earlier sections of this comment, individuals learn to find their way, not through the establishment of time-independent knowledge attained from a birds-eye view, but through the temporal ordering of vistas within a particular terrestrial expanse. This means that wayfinding, as with other situated skills, is not separable from the path of observation, which as an aside, is why there is a difference between telling someone where to go (*knowing about*), and actually going there for oneself (*knowing of*). There are, however, many different routes one can take to reach the same (or thereabouts) destination. This means that some routes, pragmatically speaking, are more efficient than others. For example, one may decide to take a shorter and more sheltered route when walking across a university campus to avoid getting wet on a particularly rainy day, while on a warmer, clearer day, the same person may decide to take a longer, less sheltered route to the same destination in order to enjoy the vista. What this simple example demonstrates is that the efficiency of the route is not concrete – what may be an efficient route today, may not be tomorrow for a variety of reasons. Wayfinding, after all, unfolds through the modulation of the person-environment system dynamics so as to yield a path that leads to a destination. This contextualised perspective holds that the skilfulness of any action is contingent on the circumstances from which it arises (Juarerro, 2023). Skilled wayfinding, then, demands an ongoing responsiveness to changes in environing conditions (Heft, 2013b; Ingold, 2000). It is a matter of *knowing of* one’s destination, not just *knowing about* it.

Herein lies the crux of this section relative to the implicit assumptions of Bock et al. (2024): *the contingent nature of wayfinding as a temporally structured skill draws into question the usefulness of a generalised (and/or strategy specific) cognitive process purported to guide wayfinding decisions*. The importance of this cannot be overstated, as it points us away from universalist and generalist positions of human wayfinding, instead moving us toward empirical investigation that takes seriously the socio-cultural contexts in which inhabitants dwell. In other words, how humans learn to find their way, as with all other situated skills, is by participating in socio-cultural practices nested within a particular set of environmental circumstances. Wayfinding, in short, is a pattern of skilled action that *develops in context* (Heft, 2013b). Indeed, this proposition leads to serious methodological considerations related to the study human wayfinding; considerations which may require psychological science to look beyond its confines. This, however, is an excursus we leave for another day.

## Concluding remarks

The aim of this commentary was to generate a humble point of contrast to the work of Bock et al. (2024), offering readers of *Psychological Research* with a different perspective as to how humans find their way. We opened by exploring the metatheoretical lineage of the target article, situating it within the broader interactionist framework. From there, we moved toward a transactional metatheoretical orientation, drawing on the ecological approach to visual perception to discuss wayfinding as a continuous, integrated perception-action process, distributed across the organism-environment system. In closing, we wish to stress that our comment is not to be construed as pushing a ‘better than’ narrative – that was not our intent. Rather, what we hope our comment shows, more broadly, is that metatheoretical orientation (implicitly) shapes the study of skilled action in psychological research, which in the present case, related to human wayfinding. As mentioned from the outset, the question of how humans find their way is profound. Hopefully, our comment has shown that its response is far from transparent, opening up some interesting lines of inquiry for readers of *Psychological Research* to follow up with.

## Data Availability

No datasets were generated or analysed during the current study.
